# Suture Distension of Schlemm's Canal in Canaloplasty: An Anterior Segment Imaging Study

**DOI:** 10.1155/2015/457605

**Published:** 2015-05-19

**Authors:** Livia M. Brandao, Andreas Schötzau, Matthias C. Grieshaber

**Affiliations:** Department of Ophthalmology, Glaucoma Service, University of Basel, Mittere Strasse 91, CH-4031 Basel, Switzerland

## Abstract

*Purpose*. The object of this study was to investigate the role of the suture stent regarding its impact on reduction of intraocular pressure (IOP) in canaloplasty based on the distension of the inner wall of Schlemm's canal. *Methods*. Nineteen glaucoma patients who underwent canaloplasty with successful positioning of the tensioning suture were included. The measurements were analyzed using linear mixed models, with the means adjusted to IOP, age, cup-to-disc ratio, and time of follow-up. *Results*. Mean follow-up time was 27.6 months (SD 10.5). Mean intraocular pressure (IOP) was 24.6 mmHg (SD 5.29), 13.8 (SD 2.65), and 14.5 (SD 0.71) before surgery, at 12 months, and at 36 months after surgery, respectively. 57.9% of patients had no medication at last evaluation. Differences and variations of measurements between the devices over a time of 12 months were not significant (*p* = 0.15 to 0.98). Some angles of distension associated with the suture stent inside SC were predictive for IOP reduction (*p* < 0.03 to < 0.001), but not for final IOP (*p* = 0.64 to 0.96). *Conclusion*. The angles of the inner wall of Schlemm's canal generated by the suture stent were comparable between OCT and UBM and did not change significantly over time. There was a tendency towards a greater distension of Schlemm's canal, when the difference was larger between pre- and postoperative IOP, suggesting the tensioning suture may contribute to IOP reduction.

## 1. Introduction

Surgical procedures involving Schlemm's canal (SC) herald a new era in glaucoma therapy. They target the site of maximal resistance to aqueous outflow in glaucoma that is the trabecular meshwork (TM) and the inner wall of SC with the intention to minimize serious complications of penetrating surgery related to hypotony and bleb-formation [1].

Canaloplasty is an ab externo procedure that allows 360-degree dilation of trabeculocanalicular outflow system. The value of canaloplasty has been demonstrated previously [1–3], but the role of the suture stent is still not understood. It has been postulated, although not measured, that the suture stent may lower the intraocular pressure (IOP) by stretching TM and inner wall of SC [4]. Imaging of the distension of SC by the intracanal stent may help to understand the role of the suture in canaloplasty and the impact on the surgical outcome.

In the past years, the interest has grown in visualizing in vivo the aqueous humor outflow system with high-resolution ultrasound biomicroscopy (UBM) and anterior segment optical coherence tomography (AS-OCT). Some studies evaluated the outflow pathway in healthy subjects [5–7] and in glaucomatous patients with viscocanalostomy [8, 9].

The aim of this study was to measure the distension of the inner wall of SC created by the suture stent in canaloplasty, using two different imaging devices, UBM and AS-OCT, and to evaluate the predictive role regarding IOP reduction.

## 2. Patients and Methods

This prospective, longitudinal study was performed at the Department of Ophthalmology, Glaucoma Service, University of Basel, Switzerland. Included were patients with medically uncontrolled open angle glaucoma (OAG) undergoing primary canaloplasty. The research followed the tenets of the Declaration of Helsinki. The study was approved by the local institutional ethics committee. Clinical data was collected continuously from 22 eyes (19 patients) that underwent surgery performed by one experienced surgeon with canaloplasty (M.C.G.). Eyes with a diagnosis or history of other types of glaucoma (e.g., angle closure, inflammatory, etc.) or ocular diseases that may interfere with the surgical results were excluded. Patients underwent a complete examination before and after surgery with best corrected visual acuity (BCVA), Goldmann applanation tonometry, slit-lamp anterior segment examination, and fundus biomicroscopy.

### 2.1. Surgical Technique

Canaloplasty combines the principle of nonpenetrating viscocanalostomy with a 360-degree circumferential distension of SC. The surgical technique has been described in detail earlier [10, 11]. The flexible microcatheter (iTrack-250A; iScience Interventional, Menlo Park, CA, USA) dilates SC and allows controlled injection of ophthalmic viscosurgical device (OVD) with posterior positioning of a 10-0 polypropylene within its extension. After subconjunctival anesthesia and corneal traction suture, the conjunctiva and Tenon's capsule were opened at limbus and the sclera was exposed. Superficial and deep parabolic shaped flaps were dissected as for viscocanalostomy in the superior quadrant. SC was deroofed and a trabeculo-Descemet membrane (TDM) window was created. Both ostia of the canal were dilated with OVD to facilitate the insertion of the microcatheter into the canal. During advancement of the tip, 0.5 *μ*L of OVD was injected every two hours clock to dilate the canal. After complete circumferential dilation, a 10-0 polypropylene suture (Prolene, Ethicon Inc., Switzerland) was affixed to the distal tip of the microcatheter, which was withdrawn to place the suture into the canal. The suture was locked under tension. The deep scleral flap was excised and the superficial flap closed watertight with 10-0 nylon sutures. Conjunctiva and Tenon's capsule were closed with 8-0 Vicryl sutures after restoring of the anterior chamber with balanced salt solution. Postoperative standard treatment was local steroids and antibiotic ointment 4 times a day for 4 weeks. Laser goniopuncture of the TDM window was performed, depending on the individual target pressure, to increase aqueous outflow into the scleral lake.

### 2.2. Imaging

Anterior segment images were obtained from each eye at 3, 6, 9, and 12 o'clock positions before surgery and at 1, 3, 6, and 12 months and thereafter every 6 months after surgery using the iUltrasound (iScience Interventional Inc., Menlo Park, CA, USA) and Visante OCT Model 1000 (Carl Zeiss Meditec Inc., Dublin, CA, USA). If the surgical dissection of the scleral flaps was exactly at the 12 o'clock position, the images were taken just next to the dissection site. All patients underwent OCT imaging before UBM and under controlled conditions of luminance.

Visante OCT is a noninvasive procedure with a 1.3 *μ*m superluminescent light-emitting diode. It has an optical axial resolution of 18 *μ*m and an optical transverse resolution of up to 60 *μ*m. It provides anterior segment scans at a rate up to 2048 A-scans per second. Images were obtained in a patient's upright sitting position using the high-resolution corneal protocol.

The principle of UBM is analogous to OCT with emission and reflection measures of sound instead of light. Unlike OCT, imaging is not dependent on surface elements, which prevent optimal imaging of underlying structures. The iUltrasound high-resolution imaging system features an 80 MHz transducer frequency with an axial resolution of 25 *μ*m and a lateral resolution of 50 *μ*m. It is optimized for imaging the upper 2 mm of the anterior segment of the eye, enabling visualization of the drainage system structures. Images were taken with the patient in supine position. The established imaging dimensions were field of view (FOV) of 3.5 mm and depth of 4 mm. The scan rate of 7 frames per second greatly reduces motion artifacts caused by hand movement. The self-contained probe of the UBM was placed directly on the eye using Methocel 2% (Omni Vision AG, Neuhausen, Switzerland) as interface and after instillation of anesthetic eye drops (Alcaine, Alcon SA, Rotkreuz, Switzerland).

**Figure 1:**
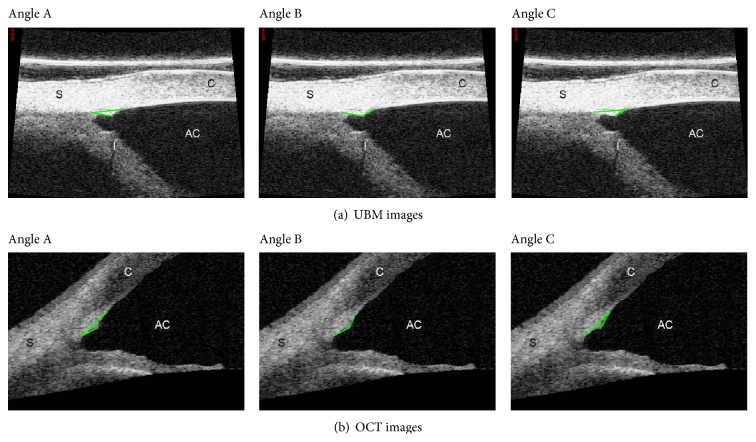
UBM imaging (a) and OCT imaging (b) of the same eye. Angles A, B, and C, respectively. The anterior chamber angle (ACA) was measured as the distance between the posterior corneal surface and the anterior iris surface. The inward distension of the canal wall (including the TM) created by the intracanal suture stent generated three angles (i.e., angle). The vectors are marked in light green. Angle A () had its apex at scleral spur with both sides extending through SC until the inner surface of corneal-scleral junction and along the distended TM. Angle B () was characterized from both sides of dilated SC and its apex at the point of maximum visible distension of the TM. Angle C () apex was at the inner corneal-scleral junction, between the scleral spur and the point of maximum distension of TM.

All postoperative images had a minimum time point of one month after surgery and magnification was adjusted to maximize visualization of angle structures. Earlier images were excluded in order to assess true tension of the suture without inference of viscoelastic material. The same examiner carried out all measurements. Degrees of anterior chamber angle at different quadrants were measured with Image J Program angle tool (version 1.44, January 2011; Rasband, W.S., Image J, U. S. National Institutes of Health, Bethesda, Maryland, USA, http://imagej.nih.gov/ij/, 1997–2011) as described in Figure 1.

### 2.3. Statistical Analysis

Descriptive statistics were used to determine continuous variables (demographic data and IOP). Linear mixed model was performed to correlate angle measurements with clinical data, as well as to assess differences between the two anterior segment imaging methods (UBM and OCT). In this model, patients were considered as random factors. The 95% confidence intervals (CI) were estimated for each variable in the model. The level of significance being considered relevant was 0.05. Statistical analyses were performed using SPSS software for Windows, version 21.0 (SPSS Inc., IBM, Chicago, IL, USA), and the R-software version 2.15.1 (R: A language and environment for statistical computing, R Foundation for Statistical Computing, Vienna, Austria, ISBN 3-900051-07-0, URL http://www.R-project.org).

## 3. Results

There was no detectable angle of the trabecular meshwork with UBM and OCT in any patients before surgery. Data of three eyes (2 patients) were excluded from the analysis because of poor image quality impeding reliable measurements. 19 eyes of 17 patients were included (Table 1). The mean follow-up time was 27.6 months (SD 10.5 months). Mean IOP was 24.6 mmHg (SD 5.29) before surgery and 15.9 mmHg (SD 7.49), 13.8 (SD 2.65), 13.8 (SD 3.91), and 14.5 (SD 0.71) at 1, 12, 24, and 36 months after surgery, respectively. The number of medications dropped from 2.47 ± 0.77 before surgery to 0.78 ± 1.08 at the last follow-up (*p* < 0.001). The surgical success for an IOP ≤ 20 mmHg with or without medication was comparable between patients with versus without laser goniopuncture (*p* = 0.764; Kaplan-Meier analysis). One patient had a second surgery to reach target IOP at 33 months. Data of this patient were analyzed up to the last follow-up before surgical revision.

A total of 144 out of 196 OCT images (73%) and 138 out of 168 UBM images (82%) were used for analysis. The minimum follow-up time was 6 months and each patient had at least two exams at different time points. The measurements were analyzed using linear mixed models, with the means adjusted to IOP, age, cup-to-disc ratio, and time of follow-up. Anterior chamber angles (ACA) were comparable between UBM and AS-OCT except for ACA at 3 and 6 o'clock position (*p* < 0.01; Table 2). Angle B at 3 o'clock and angle A at 9 o'clock position showed statistically significant differences between the exams. Regarding change over time, only two from the sixteen measured angles showed significant difference before one year (ACA 3 h and ACA 6 h) and three (angle B 3 h, angles A and B at 9 h) after one year since surgery (Table 3). A separate analysis of the measurements variation for each device in relation to the time of exam was not significant (*p* = 0.99 for OCT and *p* = 0.16 for UBM). Regarding IOP, no relationship between the distension angles of SC and absolute postoperative IOP was found (*p* = 0.96 for OCT and *p* = 0.64 for UBM). However, IOP difference between pre- and postoperative values (IOP reduction) showed some correlation with the variation of the angles measured (Table 4). For example, in UBM, ACA angles at 12 and 9 o'clock showed a strong negative relationship with IOP reduction (*p* < 0.001 and *p* < 0.05, resp.). This suggests that the narrower the anterior chamber angle, as a result of inner wall and TDM indentation by suture, the bigger the IOP reduction.  Table 5 shows the angle variation for each exam after one year from surgery. In addition, mean values of angles (A, B, or C) both per quadrant and 360° were calculated (Table 6).

## 4. Discussion

The principle of canaloplasty is to reestablish the internal physiologic pathway by circumferential viscodilation and tensioning suture of SC. There is wide evidence for canaloplasty to reduce IOP substantially to the low to mid teens with few complications [1–3]. However, little is known about the IOP lowering mechanisms; the suture stent has been postulated to be one of the factors contributing to IOP reduction.

In a multicenter study group, the distension of SC in canaloplasty was examined by UBM in a cross-sectional and qualitative way [12, 13]. A grading scale was used for the amount of distension to correlate with IOP. In the one-year analysis [12], eyes with minimal or no canal distension showed a mean decrease in IOP of 24% whereas eyes with a discernible distension of SC had a mean decrease in IOP of 40%. At two years, mean IOP decreased from 22.9 ± 3.6 mmHg to 15.7 ± 3.1 mmHg (32%) in the eyes with discernable distension [13]. The amount of IOP reduction in our study is in agreement with previous studies; IOP decreased from 24.6 ± 5.29 mmHg to 13.8 ± 2.65 mmHg at 12 months and 14.5 ± 0.71 mmHg at 36 months with an average IOP drop of 44% and 41%, respectively.

In combined procedures with cataract surgery [14], a lower mean IOP was observed in the group with greater detectable distension compared to the group with minimal or no distension, although the difference was statistically not significant. A mean angle width of 29.4 ± 14.0 degrees after surgery and a mean SC distension angle of 14.4 ± 9.5 degrees were reported in the noncombined surgery arm of the study. In a recent publication [15] using 50 MHz and 80 MHz UBM devices, measurements were compared with a subjective grading scale of the observed amount of distension after canaloplasty. IOP decreased from 23.2 ± 4.5 mmHg to 17.0 ± 3.5 mmHg postoperatively. Eyes with an overall qualified success (postoperative IOP below 21 mmHg with or without medication) showed some correlation with SC distension (*r* = 0.64). Eyes with higher grade of canal distension showed lower IOP values, although the study sample was relatively small. In all the above-mentioned studies, the visible distension was averaged between different quadrants.

Differently from previous studies, we evaluated quantitatively the amount of SC distension at different time points. Furthermore, multiple angles (“triangle” of angles) generated by the suture stent were taken into account since the suture stent may not always be placed exactly at the same location of SC even in the hands of experienced surgeons. All three angles may change accordingly if the suture is placed more anteriorly towards Schwalbe's line. The measurements of the devices (OCT or UBM) were overall comparable and did not change significantly over time in each imaging device. In terms of IOP reduction, the results were not conclusive although there was a tendency towards a greater distension of SC, when the IOP difference was larger. Accordingly, the smaller the anterior chamber angle, the bigger the IOP reduction as measured with UBM. Although one may expect that suture tension is equally distributed along the 360° circumference, only a few quadrants (two in UBM and one in OCT out of the four quadrants positions) showed a statistical correlation.

Regarding anterior segment imaging technique, both UBM and OCT provide good image quality of the inner wall distention [16, 17]. Though we did not prove one to be superior to the other, OCT may have some advantages as being more comfortable for the patient (i.e., sitting position, absence of coupling medium, and less time consumption) and having the possibility to reproduce images at the same specific localization observing anatomical structures and the angle of the light beam [7, 18]. In contrast, UBM exam needs topical anesthesia, the patient in supine position, and more importantly an experienced examiner. We cannot exclude that some images were influenced by direct contact of the UBM probe with the eye despite great care and experience. Further, laser goniopuncture did not change the measured angles since it only created holes in the window of the TDM at the site of scleral dissection.

There are numerous reasons why we did not find any strong correlation between the distension of the suture stent and IOP in this study, and the following conclusions drawn are a kind of speculatives as this is the first study, evaluating distension in canaloplasty over time. First, true suture tension cannot yet be measured during surgery but was judged by the surgeon as the indentation of the TDM by the suture stent. Second, the amount of SC distension observed by UBM and OCT is a surrogate for applied tension and indirectly for outflow facility. In addition, the ideal suture tension is not known to achieve the lowest IOP and may vary among individuals. Too little tension may not provide an adequate reduction in outflow resistance whereas too much tension may close off the intertrabecular space potentially increasing resistance. Third, the suture stent may provide persistent effect on the inner wall of SC and increased outflow of aqueous without detectable distension. Trabecular meshwork cells hence may sense mechanical induced structural changes [19, 20] and respond to it by increasing matrix metalloproteinase (MMP) activity or levels [20–23], leading to reduced resistance to outflow [24, 25]. Thus, it is likely that biochemical pathways may act in concert with the purely stretching mechanism of the tensioning suture in canaloplasty. This may explain in part why there was no correlation between distension of SC and IOP or in other words why eyes with the same IOP level had various distensions. Forth, other postulated mechanisms comprise the improved outflow created by the TDM window and scleral lake; the 360-degree viscodilation of SC with additional microdisruption of walls [2] was not addressed.

## 5. Conclusion

Canaloplasty is a bleb-independent procedure that combines the principles of nonpenetrating viscocanalostomy with a 360° circumferential distension of SC to enhance aqueous humor drainage through the physiological outflow system. Suture distension remained stable over the time period observed and showed some correlation with IOP reduction, but not with final IOP. Thus, the effects of the suture tension on IOP are yet not fully understood, and further studies are needed to elucidate the role of the suture stent in canaloplasty.

## Figures and Tables

**Table 1:** Demographic data of 19 eyes (17 patients) and *p* values.

Age (years)	71.3 ± 7.29	
Sex (female/male)	10/7	
Diagnosis		
POAG	14	
PEX	5	
Eye (right/left)	13/6	
Cup-disc ratio	0.78 ± 0.16	
BVCA		*p* = 0.38
Preop.	0.76 ± 0.22	
Last visit	0.73 ± 0.19	
IOP (mmHg)		*p* < 0.001
Preop.	24.6 ± 5.29	
6 months	13.2 ± 3.40	
12 months	13.8 ± 2.65	
24 months	13.8 ± 3.91	
36 months	14.5 ± 0.71	
Follow-up time (months)	27.6 ± 10.5	
MD		*p* = 0.05
Preop.	9.41 ± 5.63	
Last visit	7.29 ± 5.97	
Goniopuncture (yes/no)	8/11	
Number of antiglaucoma medications		*p* < 0.001
Preop.	2.4 ± 0.77 (1–4)	
Last visit	0.7 ± 1.08 (1–3)	

**Table 2:** Results of mixed effects models^*∗*^ comparing UBM and OCT measurements.

Angle	Difference of means (UBM − OCT)	95% CI	*p* value
ACA 3 h	−6.38	−11.15/−1.60	0.008
ACA 6 h	−7.07	−11.93/−2.20	0.004
ACA 9 h	−0.57	−5.47/4.33	0.818
ACA 12 h	4.41	−1.21/10.04	0.123

3 h			
Angle A	3.74	−1.08/8.56	0.128
Angle B	−7.66	−12.48/−2.84	0.001
Angle C	2.49	−2.33/7.31	0.310

6 h			
Angle A	2.11	−2.57/6.79	0.376
Angle B	−0.26	−4.94/4.42	0.913
Angle C	−3.26	−7.94/1.42	0.171

9 h			
Angle A	5.67	0.81/10.52	0.022
Angle B	−4.44	−9.30/0.42	0.073
Angle C	2.99	−1.86/7.85	0.226

12 h			
Angle A	2.77	−2.52/8.05	0.304
Angle B	−4.51	−9.75/0.73	0.091
Angle C	0.84	−4.40/6.07	0.754

^*∗*^Means were taken from a regression model and adjusted for age, pre- postoperative IOP difference, time of exam, and C/D ratio.

**Table 3:** Results of mixed effects models^*∗*^ comparing UBM and OCT values and time progression.

Angle	Difference of means (UBM − OCT) before/after one year	*p* value before/after one year
ACA 3 h	−9.17/−3.28	0.004/0.367
ACA 6 h	−6.82/−7.09	0.039/0.053
ACA 9 h	2.17/−4.38	0.520/0.232
ACA 12 h	5.67/2.74	0.130/0.532

3 h		
Angle A	1.70/6.35	0.606/0.080
Angle B	−6.32/−8.73	0.055/0.016
Angle C	1.54/3.78	0.640/0.297

6 h		
Angle A	1.45/3.29	0.648/0.351
Angle B	−0.36/0.20	0.909/0.955
Angle C	−4.55/−1.31	0.153/0.711

9 h		
Angle A	3.38/8.20	0.305/0.027
Angle B	1.21/−10.84	0.713/0.003
Angle C	0.08/7.45	0.980/0.045

12 h		
Angle A	3.88/1.13	0.267/0.785
Angle B	−5.39/−2.92	0.117/0.483
Angle C	0.48/1.99	0.887/0.633

^*∗*^Means were taken from a regression model and adjusted for age, pre- postoperative IOP difference day, and C/D ratio.

**Table 4:** Results of mixed effects models^*∗*^ analysis of UBM and OCT measurements in relation to IOP reduction^*∗∗*^.

Angle	Difference of means (UBM/OCT)	SD ± (UBM/OCT)	*p* value (UBM/OCT)
ACA 3 h	−0.64/−0.64	0.76/0.72	0.097/0.083
ACA 6 h	−0.37/−1.35	0.80/0.74	0.361/<0.001
ACA 9 h	−0.86/−0.33	0.77/0.73	0.030/0.376
ACA 12 h	−1.64/−0.48	0.89/0.87	<0.001/0.282

3 h			
Angle A	−0.26/0.04	0.73/0.77	0.490/0.914
Angle B	0.62/0.35	0.73/0.77	0.095/0.368
Angle C	−0.20/−0.23	0.73/0.76	0.593/0.563

6 h			
Angle A	0.02/0.03	0.73/0.70	0.494/0.944
Angle B	0.02/−0.48	0.74/0.71	0.957/0.182
Angle C	0.28/0.13	0.74/0.70	0.460/0.726

9 h			
Angle A	−0.29/0.13	0.78/0.74	0.466/0.727
Angle B	0.45/0.09	0.78/0.74	0.262/0.806
Angle C	0.11/0.13	0.78/0.75	0.773/0.723

12 h			
Angle A	0.17/−0.04	0.75/0.98	0.934/0.651
Angle B	−0.18/0.15	0.75/0.95	0.630/0.751
Angle C	0.09/0.12	0.75/0.95	0.808/0.798

^*∗*^Means were taken from a regression model and adjusted for age, time of exam, and C/D ratio.

^*∗∗*^IOP reduction: difference between preoperative and postoperative IOP.

**Table 5:** Results of mixed effects models^*∗*^ analysis of UBM and OCT measurements in relation to time^*∗∗*^.

Angle	Difference of means (UBM/OCT)	±SD	*p* value (UBM/OCT)
ACA 3 h	−1.15/−7.28	7.17/6.42	0.754/0.026
ACA 6 h	−2.10/−2.07	7.34/6.43	0.573/0.527
ACA 9 h	−8.21/−1.86	7.25/6.66	0.026/0.583
ACA 12 h	−2.15/0.25	8.42/7.67	0.616/0.949

3 h			
Angle A	−1.62/−6.45	6.90/6.78	0.644/0.062
Angle B	7.27/9.51	6.90/6.78	0.039/0.006
Angle C	−2.93/−5.34	6.90/6.78	0.405/0.122

6 h			
Angle A	2.57/0.71	6.90/6.36	0.465/0.825
Angle B	−0.75/−1.32	6.90/6.37	0.830/0.683
Angle C	2.13/−0.94	6.90/6.37	0.510/0.771

9 h			
Angle A	−0.44/−5.30	7.11/6.75	0.903/0.123
Angle B	−3.60/8.41	7.11/6.75	0.320/0.014
Angle C	6.61/−0.80	7.11/6.75	0.068/0.816

12 h			
Angle A	−2.75/−0.41	7.51/7.67	0.473/0.916
Angle B	1.77/−1.12	7.51/7.57	0.644/0.772
Angle C	3.67/1.75	7.52/7.57	0.337/0.650

^*∗*^Means were taken from a regression model and adjusted for age, time of exam, and C/D ratio.

^*∗∗*^Time: after one-year follow-up.

**Table 6:** Angle measurements values (means).

Exam (clock position)	Means ± SD	
	OCT (°)	UBM (°)
“3-hour”		
Angle A	15.3 ± 6.9	19.2 ± 6.5
Angle B	147.6 ± 14.4	140.0 ± 15.7
Angle C	15.9 ± 9.4	18.4 ± 8.1
“6-hour”		
Angle A	16.7 ± 6.3	18.8 ± 6.4
Angle B	143.5 ± 13.8	143.2 ± 14.6
Angle C	19.3 ± 9.1	16.0 ± 9.7
“9-hour”		
Angle A	15.5 ± 6.1	21.2 ± 4.6
Angle B	145.0 ± 11.1	140.6 ± 10.6
Angle C	15.49 ± 8.9	18.5 ± 8.3
“12-hour”		
Angle A	15.92 ± 5.3	19.1 ± 5.3
Angle B	145.0 ± 12.8	140.7 ± 14.3
Angle C	19.0 ± 8.9	19.5 ± 9.7
Mean of 4 hours-positions/eye		
Angle A	16.0 ± 4.6	19.5 ± 4.5
Angle B	145.2 ± 9.8	141.4 ± 10.5
Angle C	17.5 ± 5.7	17.95 ± 6.9
